# Elastic mucus strands impair mucociliary clearance in cystic fibrosis pigs

**DOI:** 10.1073/pnas.2121731119

**Published:** 2022-03-24

**Authors:** Maria I. Pino-Argumedo, Anthony J. Fischer, Brieanna M. Hilkin, Nicholas D. Gansemer, Patrick D. Allen, Eric A. Hoffman, David A. Stoltz, Michael J. Welsh, Mahmoud H. Abou Alaiwa

**Affiliations:** ^a^Department of Internal Medicine, Pappajohn Biomedical Institute, Roy J. and Lucille A. Carver College of Medicine, University of Iowa, Iowa City, IA 52242;; ^b^Department of Pediatrics, Pappajohn Biomedical Institute, Roy J. and Lucille A. Carver College of Medicine, University of Iowa, Iowa City, IA 52242;; ^c^Department of Radiology, Roy J. and Lucille A. Carver College of Medicine, University of Iowa, Iowa City, IA 52242;; ^d^Department of Biomedical Engineering, University of Iowa, Iowa City, IA 52242;; ^e^Department of Molecular Physiology and Biophysics, Roy J. and Lucille A. Carver College of Medicine, University of Iowa, Iowa City, IA 52242;; ^f^HHMI, University of Iowa, Iowa City, IA 52242

**Keywords:** lung, epithelia, cystic fibrosis, mucus, submucosal gland

## Abstract

Defective mucociliary transport contributes to infections and inflammation that destroy cystic fibrosis (CF) lungs. To better understand mechanisms that impair mucociliary transport, we tracked insufflated microdisks with X-ray computed tomography in spontaneously breathing CF pigs. Aerosolized saline increased microdisk movement toward the larynx. Surprisingly, forward progression was repeatedly interrupted as microdisks abruptly recoiled. Thus, although saline increased motion, it had minimal effects on microdisk clearance. To test if abnormally elastic mucus strands were responsible, we aerosolized Tris-carboxyethyl phosphine (TCEP), a reducing agent that breaks disulfide bonds linking mucins. TCEP largely eliminated retrograde movement and increased microdisk clearance. Ex vivo studies confirmed that TCEP broke CF mucus strands, unleashing them from submucosal gland ducts, and cilia carried them rostrally. These findings emphasize the role of abnormally elastic submucosal gland mucus strands in impairing CF mucociliary clearance and suggest that disrupting mucus strands may improve mucociliary clearance in CF.

## Significance

In many lung diseases, increased amounts of and/or abnormal mucus impair mucociliary clearance, a key defense against inhaled and aspirated material. Submucosal glands lining cartilaginous airways secrete mucus strands that are pulled by cilia until they break free from the duct and sweep upward toward the larynx, carrying particulates. In cystic fibrosis (CF) pigs, progressive clearance of insufflated microdisks was repeatedly interrupted as microdisks abruptly recoiled. Aerosolizing a reducing agent to break disulfide bonds linking mucins ruptured mucus strands, freeing them from submucosal gland ducts and allowing cilia to propel them up the airways. These findings highlight the abnormally increased elasticity of CF mucus and suggest that agents that break disulfide bonds might have value in lung diseases with increased mucus.

Mucociliary transport (MCT) is an innate defense mechanism that protects lungs from inhaled and aspirated material (1–3). MCT depends on mucus, which traps particulates and pathogens, and motile cilia, which protrude from epithelia lining the airways and by their beating propel mucus up the airways and out of the lung. In the cartilaginous airways of humans, pigs, and likely other large mammals, submucosal glands (SMGs) are required for normal MCT (4, 5). SMGs expand the number of fluid- and mucus-producing cells beyond that in surface epithelia and secrete much of the airway mucus (5–7). The major structural component of mucus is mucins, and SMGs produce the mucin MUC5B, which is key for MCT (8–10). SMGs secrete mucus under basal conditions, and when the respiratory tract is challenged, they respond to neurohumoral signals by secreting copious amounts of mucus (5, 11–13).

Mucus emerges from SMG ducts onto the airway surface in the form of mucus strands (10, 14–18). Beating cilia pull the mucus strands; they stretch, eventually break, and are then swept upward toward the larynx (14–16). Elasticity dominates the biophysical properties of the mucus (19, 20). Reducing agents break the disulfide bonds that link mucins and thereby disrupt the integrity of mucus strands (14, 21–24). As a result, when reducing agents were applied to the airway surface liquid (ASL), the mucus emerging from SMG ducts immediately fragmented, failed to generate strands, and thereby prevented normal MCT (14). Thus, mucus strands secreted from SMGs make key contributions to MCT. Consistent with this conclusion, when pigs lack the SMGs that produce mucus strands, MCT is disrupted (4).

MCT is defective in cystic fibrosis (CF), a life-threatening genetic disease caused by mutations in the gene encoding the cystic fibrosis transmembrane conductance regulator (CFTR) anion channel (2, 25, 26). MCT is impaired, in part, by the altered biophysical properties of the mucus produced by CF SMGs (10, 15, 16, 20). Defective SMG mucus is apparent even before birth; histopathologic examination of lungs from human fetuses with CF revealed abnormal lamellated Periodic acid-Schiff positive material obstructing the lumen and ducts of airway SMGs (27, 28). Similar changes were observed in deceased infants with CF, although inflammation and infection occurring after birth may have contributed (17, 28–30). Studies in CF pigs and sheep immediately after birth also reveal mucus accumulation in SMG ducts and acini (10, 15, 16, 31). Mucus accumulation in SMGs also appears in CF ferrets and rats once they develop SMG glands postnatally (32, 33) and in older CF rabbits in Bowman glands of the olfactory epithelium (34). The abnormal mucus and impaired MCT contribute to the recurrent airway infections and inflammation in CF.

Unlike mucus strands secreted from wild-type SMGs, mucus strands from CF SMGs often fail to break after emerging from their ducts. As a result, they accumulate and prevent particles from moving up the airways (10, 14–16). Defective HCO_3_^−^ and Cl^−^ secretion in SMGs are responsible for the abnormal mucus strands (10, 15); without CFTR function, SMG liquid becomes abnormally acidic, and the protein concentration increases (20, 35). These abnormalities produce mucus with increased elasticity and tensile strength and reduced breakage when mucus strands are stretched (15, 20). In freshly excised non-CF airways, removing HCO_3_^−^ and inhibiting Cl^−^ secretion with bumetanide reproduces CF-like conditions so that mucus strands emerging from SMGs often fail to break loose from the ducts and accumulate on the airway surface, preventing normal MCT (10, 15).

The reducing reagent Tris-carboxyethyl phosphine (TCEP) breaks mucus strands (14, 21, 22). Because abnormal mucus strands impair MCT in CF, we asked if aerosolized TCEP would enhance MCT in CF pigs. The answer was of interest because TCEP has the opposite effect in non-CF pigs, decreasing MCT (14). We also wished to know the answer because reducing agents are being investigated as a potential therapy for CF (23, 24). However, before directly addressing this question, we needed to assess the effect of aerosolized saline, which is used to deliver the TCEP. We were encouraged to do that because in a previous in vivo study, we found that aerosolizing 0.5 mL of saline to non-CF pigs increased MCT (14). In other studies, isotonic physiologic salt solutions reversed the impairment in MCT induced by blocking Cl^−^ and HCO_3_^−^ secretion in porcine tracheas (36), and in humans, inhalation of isotonic saline increased mucociliary clearance in normal and asthmatic lungs (37).

Here, we took advantage of a model of CF in pigs, which like humans have abundant SMGs and replicate CF abnormalities. We also used a computed tomography (CT) imaging method with high spatial and temporal resolution that reveals the trajectories of individual microdisks as they are carried by mucus strands within intrapulmonary airways.

## Results

### Despite Aerosolizing Saline and Cholinergic Stimulation, MCT Was Impaired in CF Pigs.

**Fig. 1.**
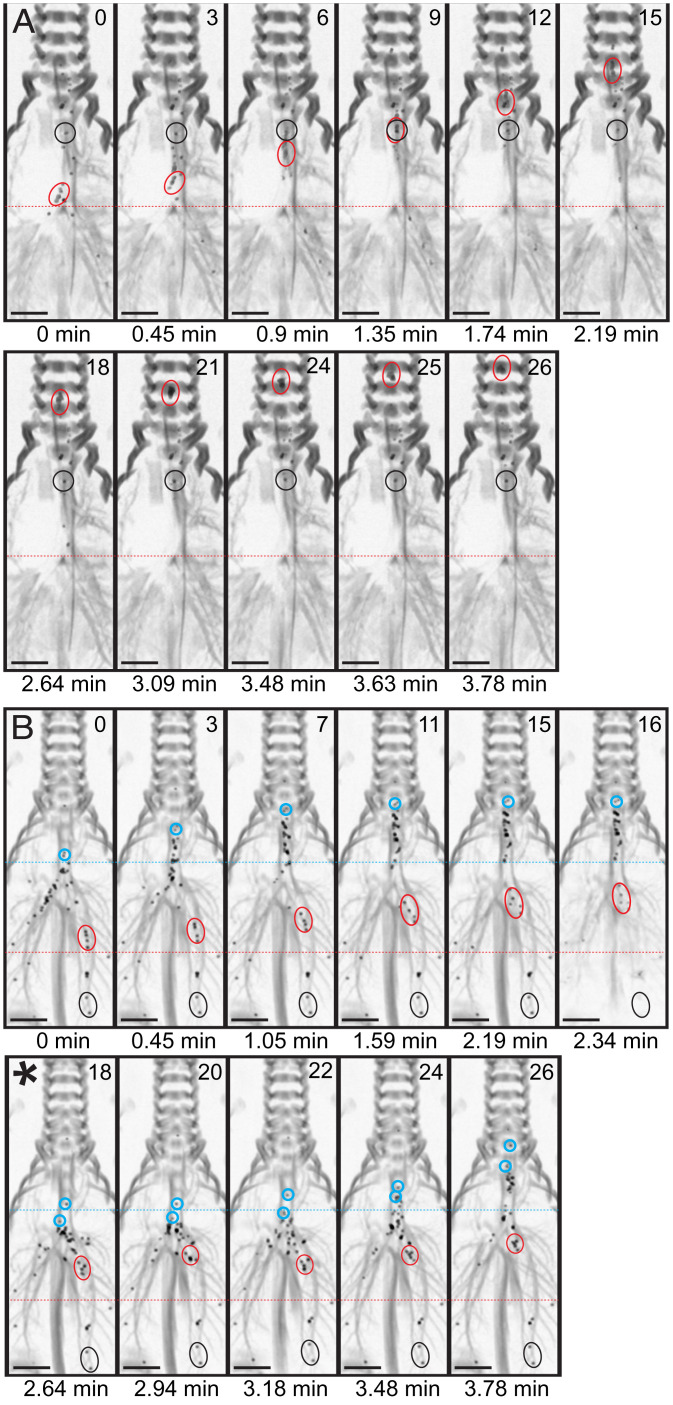
Aerosolized saline induces abrupt retrograde movement of microdisks in CF airways. Maximal intensity projection of a dynamic CT scan from a CF pig in coronal reconstruction, showing microdisk deposition and movement in the airways. (*A*) Basal conditions. Black circles indicate a cluster of nonmoving microdisks; red circles indicate a cluster of moving microdisks. (*B*) After IV methacholine and intratracheal saline. Black circles indicates a cluster of nonmoving microdisks; red and blue circles indicate clusters of moving microdisks that are transported ∼2 cm from their original location in ∼2.5 min before they abruptly (∼8.5 s) recoiled backward in a single frame (asterisk). Real time in minutes and frame number are indicated for each panel. Images were inverted to enhance contrast between microdisks and airway tree shadow. (Scale bar = 1 cm.).

**Fig. 2.**
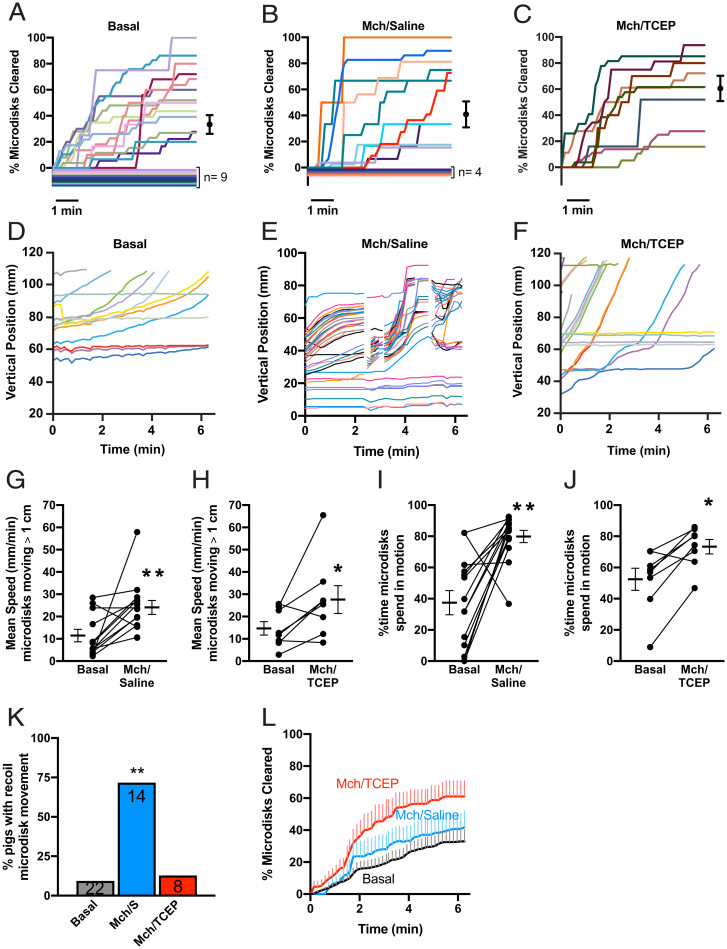
Aerosolized saline increases microdisk movement, but recoil transport impairs their clearance from CF airways. (*A*–*C*) Percentage of microdisks cleared, stratified by experimental condition. Each animal is depicted with a unique color. Lines with 0% clearance are nudged for visualization purposes. Conditions include (*A*) basal unstimulated (mean = 33 ± 7%, *n* = 22), (*B*) simultaneous IV methacholine and inhaled saline (mean = 42 ± 10%, *n* = 14), and (*C*) simultaneous IV methacholine and inhaled TCEP (mean = 61 ± 10%, *n* = 8). Dots and whiskers are mean ± SEM. (*D*–*F*) Vertical position of microdisks as a function of time in representative experiments. (*D*) Under basal conditions, most microdisks move slowly, and some are stationary. (*E*) After simultaneous treatment with IV methacholine and inhaled saline, microdisk velocity was increased compared to basal conditions. Discontinuous traces indicate times when microdisks abruptly moved backward (Movie S2). (*F*) After IV methacholine and inhaled TCEP, microdisks advanced through the airway without apparent recoil reversals. (*G* and *H*) Average speed of moving microdisks. Each set of data points and connecting lines is from a different pig. Lines and error bars represent mean ± SEM. (*I* and *J*) Percentage of time microdisks spend in motion. Each set of data points and connecting lines is from a different pig. Some data points overlap. Lines and error bars represent mean ± SEM. (*K*) Percentage of pigs with observed recoil movements. Bars represent the percentage of pigs observed to have recoil. Number of experiments per condition are given in the bars. (*L*) Mean percentage of microdisks cleared from the field. Lines represent mean and vertical lines represent SEM for basal (black), IV methacholine and inhaled saline (blue), or IV methacholine and TCEP (red). Mch: Methacholine; S: Saline; *P < 0.05 and **P < 0.01 by paired *t* test.

To measure MCT, we insufflated radiodense tantalum microdisks (350 µm) into newborn CF pigs breathing spontaneously through the nose (Fig. 1*A* and Movie S1). We immediately obtained dynamic volumetric X-ray CT scans every 8.6 s for 6.3 min (14, 15, 38). We determined the positions and movement of the microdisks at each time point. We determined the percentage of microdisks that cleared the lung, i.e., reached the larynx, for individual pigs under basal conditions (Fig. 2*A*). By the end of the 6.3-min scanning period, the average number of microdisks clearing the lung was 33 ± 7% (mean ± SEM). However, there was substantial variability: in 41% (*n* = 9) of the pigs, no microdisks cleared from the lung, and in 5% (*n* = 1) of the pigs, all the microdisks cleared.

We asked if aerosolizing saline at the same time as we stimulated SMGs would increase microdisk clearance. To stimulate SMG secretion, we delivered the cholinergic agonist methacholine intravenously (IV) as previously reported (14). We aerosolized 0.5 mL of saline with a droplet diameter of 30 to 100 μm, which will increase ASL volume in the trachea and large bronchi. However, saline and methacholine failed to significantly increase clearance: after 6.3 min, microdisk clearance averaged 42 ± 10% (mean ± SEM) (Fig. 2*B*). Moreover, the percentage of pigs with no microdisks cleared (29%, *n* = 4) or with all of the microdisks cleared (7%, *n* = 1) did not significantly differ from basal conditions. These results contrast with data in non-CF pigs in which aerosolized saline and IV methacholine more than doubled clearance, from 31 ± 10% to 74 ± 10% (14); for comparison, *SI Appendix*, Fig. S1, shows previously published microdisk clearance data from non-CF pigs.

### Aerosolized Saline Induced Intermittent Retrograde Microdisk Movement in CF Pigs.

Tracking the positions of individual microdisks revealed why saline and methacholine did not increase clearance. Consistent with our earlier work, microdisk transport was heterogeneous within an animal (Fig. 2*D* and Movie S1) (14, 15). Under basal conditions, some microdisks moved smoothly in the rostral direction until they cleared the airways, and some failed to move. Although aerosolized saline failed to significantly increase clearance, it markedly altered the movement of microdisks. Saline plus methacholine doubled the percentage of time that microdisks were in motion and doubled their average speed (Fig. 2 *G* and *I*). These increases parallel those that we previously observed in non-CF pigs (14).

However, in tracking the microdisks, we were surprised to find that their forward progression was abruptly interrupted as they suddenly recoiled backward, returning to near their initial position (Figs. 1*B* and 2*E* and Movie S2). This prominent behavior suggested that elastic mucus strands pulled the microdisks backward. Aerosolized saline induced this abrupt recoil movement in 71% of the CF pigs, whereas it occurred in 9% of CF animals under basal conditions (Fig. 2*K*).

The abrupt retrograde movements were not due to respiratory movements as stationary microdisks in CF pigs remained in fixed positions (Fig. 1*B*). The appearance of retrograde movement was also not due to proximal movement of microdisks from more distal parts of the lung because the CT scan of the entire lung detected no more distal microdisks. This abrupt movement was much faster than our image acquisition. Thus, it was not possible to identify the same microdisks with each recoil and backward movement. We occasionally observed that retrograde motion of some microdisks occurred simultaneously into more than one bronchus. This behavior suggests that mucus strands originating from distinct airway branches joined together in the trachea and then pulled apart when retrograde elastic forces retracted them to the bronchi of their origins.

### TCEP Eliminated Retrograde Microdisk Movement and Increased Clearance in CF Pigs.

Our earlier finding that TCEP breaks mucus strands suggested that TCEP might increase microdisk clearance in CF pigs (*SI Appendix*, Fig. S1) (14). We tested this prediction by including 10 mM TCEP in the 0.5 mL of aerosolized saline.

As we observed with saline alone, saline containing TCEP increased both the percentage of time that microdisks were in motion and their average speed (Fig. 2 *H* and *J*). However, TCEP largely eliminated the recoil movement (Fig. 2 *F* and *K*). As a result, TCEP increased the number of microdisks clearing the lung during the 6.3-min tracking period (Fig. 2 *C* and *L*). Moreover, at least some microdisks cleared the lung in all the pigs tested. These data suggest that impaired breakage of mucus strands inhibits MCT in CF pigs.

### Aerosolized Saline Induced Intermittent Retrograde Microdisk Movement.

In the studies described above, we found repeated intermittent retrograde microdisk movement when we concurrently delivered aerosolized saline and IV methacholine. However, in previous studies, delivering IV methacholine alone without aerosolized saline did not induce the recoil (14, 15). This difference suggested that it was the increase in airway liquid from saline aerosolization that allowed microdisks to recoil back in CF pigs.

**Fig. 3.**
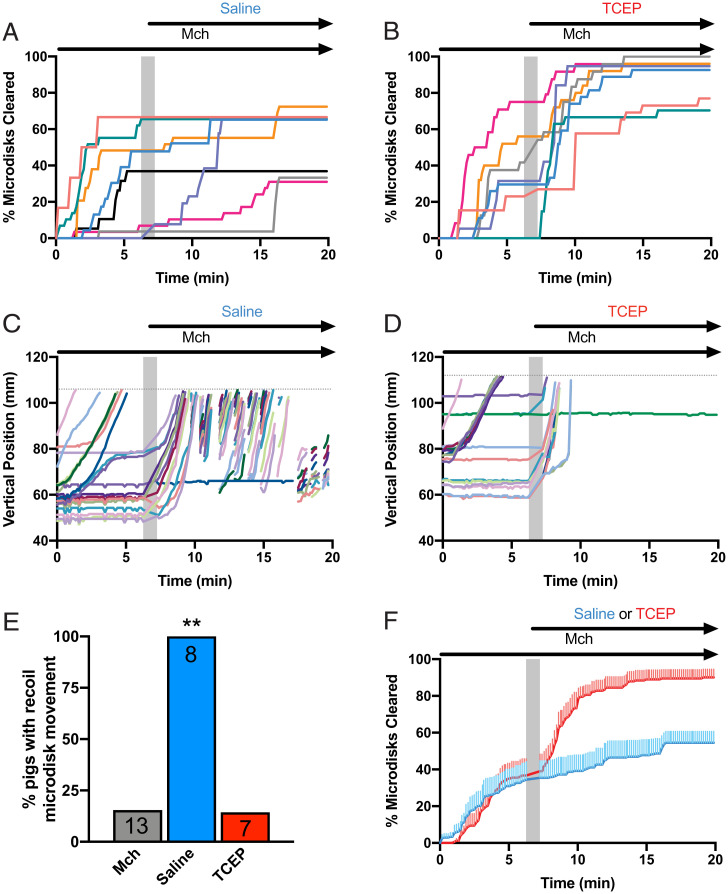
TCEP largely eliminates recoil transport of microdisks and allows their clearance from CF airways. CF pigs were treated with methacholine (McH) to increase SMG secretion, trapping Tantalum microdisks. After 6 min, the pigs were treated with either inhaled saline or inhaled TCEP in saline. (*A* and *B*) Data show the percentage of microdisks cleared as a function of time. Lines represent individual experiments performed in the presence of saline (*A*) or TCEP/saline (*B*). (*C* and *D*) Data show vertical positions of microdisks as they are transported in two representative experiments in CF pigs. Each microdisk is given a unique color. Dotted line is the limit of the tracking field at the level of the larynx. (*E*) Percentage of experiments with observation of recoil movements. (*F*) Mean percentage of microdisks cleared from the field. Lines represent mean and vertical lines represent SEM for inhaled saline (blue) or TCEP in saline (red). Mch: Methacholine; gray bar represents 1 min pause in image acquisition to administer intratracheal intervention; **P < 0.01 by paired *t* test.

To test this prediction, we first stimulated SMG secretion in CF pigs with IV methacholine and recorded microdisk movement for a 6.3-min period and then aerosolized saline and recorded microdisk movement for a 12.6-min second period. During the first period, some microdisks cleared the lung, but most either did not move or failed to clear; Fig. 3*A* shows summary data for eight pigs, and Fig. 3*C* shows microdisk trajectories from one pig. In the example from one pig, aerosolized saline initiated microdisk movement (Fig. 3*C*), but most of these microdisks failed to clear the lungs because they abruptly recoiled backward. Aerosolized saline increased clearance in two of the eight pigs but had minimal effect on clearance in the other five pigs. None of the CF pigs treated with inhaled saline cleared more than 70% of the microdisks. The repeating pattern of progression and recoiling occurred in all eight pigs treated with saline (Fig. 3*E*). Moreover, compared to the end of the first period, the average increase in clearance did not reach statistical significance (*P* = 0.063, Wilcoxon signed-rank test).

### Aerosolized Saline Containing TCEP Eliminated Retrograde Microdisk Movement and Increased Clearance.

To further test whether elastic mucus strands prevented microdisk clearance, we included TCEP in the saline delivered at the start of the second tracking period. We were also interested in this experimental protocol because it might more closely reflect a therapeutic approach of delivering a reducing agent to disrupt mucus strands that are already attached at SMG ducts. TCEP rapidly increased the clearance of most of the microdisks (Fig. 3*D*). After TCEP, five of seven pigs cleared >70% of the microdisks (Fig. 3*B*). Moreover, TCEP largely eliminated retrograde movement (Fig. 3*E*). Fig. 3*F* shows the average time course of clearance for all the pigs during the first period with methacholine alone and then during the second period with saline ±TCEP. Compared to the basal period and to saline alone, saline plus TCEP improved microdisk clearance.

### TCEP Broke Mucus Strands in Ex Vivo CF Trachea Immersed in Saline.

The finding that retrograde particle movement was eliminated by TCEP in vivo led us to additional studies ex vivo. We excised airways from CF pigs and immersed them in saline. We stimulated SMG secretion with methacholine, added fluorescent nanospheres to label the mucus, and recorded mucus movement with confocal microscopy as previously described (14, 15, 38).

**Fig. 4.**
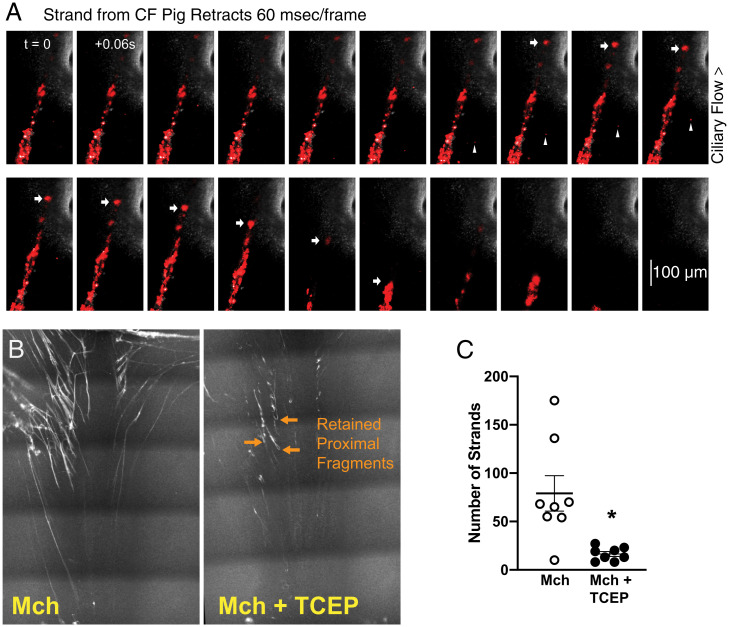
TCEP breaks mucus strands in ex vivo CF airway. (*A*) Sequential confocal microscopy images (60 ms/frame) depict a mucus strand from a CF pig, labeled with red fluorescent nanospheres. Ciliary transport directs free particles toward the top of the image (arrowheads). Arrows show the position of the distal portion of a mucus strand. The distal end of the strand spontaneously retracts. See also Movie S3. (*B*) Confocal microscopy images show stationary mucus strands before and after treatment with TCEP from a representative experiment. (*C*) Number of stationary strands in the microscopy field before and after treatment with TCEP. Mch: methacholine; open circles: before treatment with TCEP; filled circles: after treatment with TCEP; *P < 0.05 by paired *t* test.

Mucus strands stretched out over the airway surface, pulled by beating cilia. We found that mucus strands sometimes suddenly retracted in the opposite direction with a recoil speed greater than the speed of forward flow; Fig. 4*A* and Movie S3 show an example. These results are consistent with our in vivo observations. To test if TCEP would disrupt strands that were attached to SMG ducts, we examined tracheas using a panoramic confocal imaging and an averaging technique that highlights stationary mucus strands (15). We found that TCEP broke the mucus strands, generally in close proximity to SMG duct openings (Fig. 4 *B* and *C* and Movie S4). As a result, the mucus fragments flowed to the edge of the tissue. Recent data suggest that TCEP alters the rheological properties of mucus collected from airways of patients with asthma (39). We measured the microrheology of CF mucus using single-particle tracking. Consistent with earlier reports of viscoelastic properties of CF mucus (40–43), elasticity predominated. TCEP reduced the elastic properties notably at lower frequencies (*SI Appendix*, Fig. S2).

## Discussion

Our data, together with previous reports, indicate that defective bicarbonate and chloride secretions in CF SMGs produce strands of mucus with abnormal biophysical properties (10, 15, 19, 20). As a result, they often fail to break free from SMG ducts. A striking demonstration of this defect came after saline aerosolization when we found that following progressive movement toward the larynx, mucus strands sometimes abruptly and rapidly recoiled backward. Retraction occurred both in vivo and ex vivo and prevented clearance.

Because retraction was uncommon under basal conditions, we considered how saline administration might induce repetitive retractions. The recoil depended on anchoring one end of the mucus strands at the SMG duct, on beating cilia that pull and stretch the mucus strands rostrally, and on mucus strands with the elasticity and tensile strength to reduce the tendency to break (15, 20). We speculate that when saline increases ASL volume, cilia more effectively initiate mucus strand movement. We speculate that saline increases ASL volume, slightly lifting the mucus strands from the epithelial surface. As a result, cilia more effectively initiate their movement. When the elastic recoil force of the mucus strand exceeds the stretching force generated by cilia, the strand snaps back toward its anchor at the mouth of the SMG duct. When combined with the abnormal elastic force of CF mucus, frictional force from inspiratory airflow might trigger recoil. However, inspiratory airflow itself was not sufficient because it did not cause mucus strand recoil in non-CF pigs treated with saline (14). We note that abrupt retractions also occurred ex vivo in trachea submerged in saline. It was less common there, likely because the abundance of saline minimizes the interaction between mucus strands and cilia and thus there is likely less stretching and tension in strands. These results suggest that saline had a beneficial effect by increasing the movement of mucus strands; adding TCEP had no additional effect on the fraction of time that microdisks were in motion or their speed. The beneficial effect of TCEP was that it enhanced the effect of saline by increasing the breakage of mucus strands from their anchor point and thereby increasing their clearance.

This study has several advantages and limitations. First is the model. Like humans, pigs have SMGs in proximal airways, and the porcine CF model reproduces defects of human CF (26, 44). In addition, animals were spontaneously breathing with normal airway humidity. Because we studied CF pigs as newborns, they exhibit the respiratory host defense defects of CF but lack the infection, inflammation, and airway remodeling that can confound results (35, 45). Previous studies have described mucus viscosity, adhesivity, cohesivity, and elasticity and reported increased elasticity in CF sputum (46–48). However, sputum also contains products of infection and inflammation, including DNA, that may contribute to its mechanical properties (40–43). A limitation of studying newborns is that their neurohumoral responses may differ from those of adults.

Second is the methods. By using CT scans with high temporal and spatial resolution, we were able to identify discrete intrapulmonary airways, ascertain trajectories of individual microdisks, determine whether particles are stationary or moving, learn when they clear from the lung, and discover the surprising recoil behavior. The resolution inherent in the methods also revealed surprising variability in MCT and clearance, including at the level of individual microdisks, between individual animals, and in the response to the interventions. Discovering this variability would have been impossible in previous studies of CF. At present, we cannot identify experimental or specific biological factors responsible for the variability. However, we do note that the clinical pulmonary phenotype exhibits broad variation in people with CF and CF pigs (26, 49). We used microdisks because we could detect them by CT. A limitation of using microdisks is that they are larger than many inhaled pathogens and particulate material. However, we note that they are in the range of some aspirated particles (50). Moreover, use of the microdisks allowed us to identify the behavior of mucus strands, which bind microspheres that are the same size as and nanospheres that are smaller than inhaled pathogens (14–16).

An advantage and also a limitation of this study is that we focused on one component of mucociliary clearance: the contribution of mucus from SMGs. Loss of CFTR may also disrupt other aspects of mucociliary clearance (15, 17, 18). CFTR is expressed in surface epithelia of proximal and distal airways, and dysfunction at those sites likely also contributes to CF lung disease (51). For example, mucus produced by goblet cells in surface epithelia or in small airways might also be defective (52).

Aerosolized saline increased microdisk movement in both non-CF and CF airways. Whereas saline increased clearance in non-CF airways (14), the increase in clearance was minimal in CF airways. These results suggest that increasing ASL volume may be beneficial but on its own produces less than optimal mucociliary clearance. Our data suggest that aerosolized saline could improve the clearance of particles transported on mucus strands that are not attached to the SMG duct; saline increased clearance once TCEP severed strands. It is also possible that saline alone might improve clearance in distal airways that lack SMGs, i.e., beyond ∼10 generations. Hypertonic saline has additional potential activities that might contribute to its benefits, including causing neuronal activation and cough, and the inclusion of coadministered β-adrenergic agonists (53, 54). These results may be consistent with previous studies showing clinical benefit after people with CF inhale hypertonic saline (55, 56).

The development of highly effective CFTR modulators has changed the clinical outcome of CF airway disease (57, 58). A majority of people with CF carry mutations that are targets of these drugs. While these newer therapeutics improve lung function and mucociliary clearance, they do not completely eliminate chronic bacterial infection, airway inflammation, and remodeling (57, 58). In addition, some people with CF have CFTR mutations that are not amenable to the effects of these drugs. Therefore, additional treatments are still needed. Our results highlight the importance of SMGs in impairing mucociliary clearance and raise questions about whether targeted therapy should be tailored to improve function and modify mucus of SMGs. Whereas modulators improve CFTR function throughout the lung because it is delivered systemically, several potential inhaled therapies target airway surface epithelia and not SMGs. These considerations raise the question, Will correcting defective MCT in surface epithelium alone be sufficient to restore normal airway function? Because multiple host defense defects cause CF airway disease, we suspect that correcting some, but not all, defects will yield clinical benefit. Thus, improving function at any site might prove beneficial.

Our results suggest that delivering an agent to cleave mucus strands might have therapeutic value by releasing anchored mucus and permitting removal of accumulated mucus from the lungs. Here we used TCEP to test a hypothesis; however, it is not an approved drug, and we were not testing its therapeutic potential. *N*-acetyl cysteine is approved for inhaled use and might potentially have benefit, as might other agents that cleave disulfide bridges by chemical reduction or thiol exchange. A caution to this approach is the observation that in non-CF lungs, TCEP impaired mucociliary clearance because it prevented development of mucus strands (14). Thus, we speculate that delivering a reducing agent intermittently to CF airways might improve mucociliary clearance by removing accumulated mucus, whereas continuous cutting of mucus strands might hinder clearance. In addition, finding that the disease state of airways markedly influenced response to TCEP might explain apparent discrepancies in the literature for studies done in a variety of diseases, including asthma, Chronic Obstructive Pulmonary Disease, interstitial pulmonary fibrosis, and bronchiectasis. In some studies, thiol-derivative reducing agents improved lung function, whereas in other studies, it showed no effect (24, 59–64).

This work together with our prior studies portray an essential role for mucus strands secreted from SMGs. In normal airways, mucus strands are required to transport large particles out of large airways. Disrupting the architecture of these strands with reducing agents impairs MCT. In CF airways, mucus strands are abnormally elastic and fail to break loose from SMG ducts. Disrupting these strands with reducing agents improves MCT. These studies also provide a framework to assess abnormalities in other diseases with abnormal MCT, such as chronic obstructive pulmonary disease, primary ciliary dyskinesia, asthma, or idiopathic pulmonary fibrosis (2, 65–67).

## Methods

### Animals.

Newborn CF pigs were obtained from Exemplar Genetics. We studied male and female pigs 8 to 15 h after birth. Sedation was with ketamine (20 mg/kg, instramuscular [I.M.]; Phoenix Pharmaceutical, Inc.) and acepromazine (2 mg/kg, I.M.; Phoenix Pharmaceutical, Inc.) or xylazine (2 mg/kg, I.M.; Lloyd), and anesthesia was maintained with IV dexmedetomidine (10 µg/kg/h; Accord Healthcare, Inc.). Euthanasia was with IV Euthasol (Virbac).

### Delivery of Agents into the Airways.

We measured MCT in vivo before and after stimulating SMGs with IV methacholine. To understand the role of mucus strands in MCT in CF pigs, in some experiments, we aerosolized TCEP or saline vehicle concurrently with methacholine and acquired CT scans (saline, *n* = 14; TCEP, *n* = 8). In other experiments, we stimulated with IV methacholine to induce strand formation and then aerosolized TCEP or saline a few minutes later to look for the effect of TCEP on preformed strands (saline, *n* = 8; TCEP, *n* = 7). All aerosolized interventions were done using a MadGic microsprayer.

### In Vivo MCT Assay.

To measure MCT in vivo, we used a previously described X-ray CT assay. We measured MCT by tracking tantalum microdisks (350 μm diameter × 25 μm thick; Sigma). To deliver microdisks, animals were anesthetized and briefly intubated, and microdisks were insufflated into the airways just beyond the vocal cords with a puff of air. Immediately after delivery, the tube was removed. CT scans were acquired with a high-resolution multirow detector computerized tomography scanner (Siemens SOMATOM, Force Dual Source 384-slice [2 × 192] CT scanner). Thus, 44 CT scans were obtained in a 6.3-min time interval. Microdisks were tracked over time as previously described (14). Tracking microdisks over time provided multiple instantaneous measurements of microdisk speed. From these speeds, we determined individual microdisk maximum speeds and mean speeds for microdisks that moved >10 mm. Microdisk clearance was determined by measuring whether a microdisk left the tracking field or not during the 6.3-min tracking period. The percentage of microdisks cleared was determined by dividing the number of cleared microdisks by the total number of microdisks tracked × 100%. The data were obtained by a single external observer blinded to treatment condition.

### Confocal Microscopy.

Tracheas were removed from newborn CF pigs at the time of necropsy, pinned onto dental wax, and submerged in 40 mL of HEPES-buffered saline, pH 7.4, at 37 °C. Red fluorescent nanospheres were added to the preparation as previously described. We added methacholine to a final concentration of 100 µM to stimulate glandular secretion. TCEP was added to a final concentration of 1 mM to break mucus strands. Individual mucus strands were imaged on Nikon A1R confocal microscopy using a 25× submersion objective lens. Panoramic imaging with an averaging technique was performed to identify stationary mucus strands as previously described (14, 15). We used a 4× objective lens and a moving stage to cover the entire segment of trachea. Strands were then counted by three observers who were blinded to treatment condition.

### Statistical Analysis.

Differences were considered statistically significant at *P* < 0.05. All analyses were completed in GraphPad Prism v7.0d (GraphPad Software). Data from individual animals are presented as individual data points, and mean ± SEM are indicated by bars. For pairwise comparisons, we used Wilcoxon matched-pairs signed-rank test. In Figs. 2*L* and 3*F*, because whether a microdisk clears the field or not at each time point is a binary outcome, we modeled clearance with logistic regression. We used Akaike’s method to reject the simple hypothesis that one curve fit all datasets.

### Study Approval.

The present studies in animals were reviewed and approved by the University of Iowa Animal Care and Use Committee.

## Supplementary Material

Supplementary File

Supplementary File

Supplementary File

Supplementary File

Supplementary File

## Data Availability

All study data are included in the article and/or supporting information.
