# Supplementary material for: Elastic mucus strands impair mucociliary clearance in cystic fibrosis pigs
Source: Proc Natl Acad Sci U S A. 2022 Mar 24;119(13):e2121731119. doi: 10.1073/pnas.2121731119 (PMC9060506; doi:10.1073/pnas.2121731119)
Supplement: Supplementary File [file pnas.2121731119.sapp.pdf]

Supplementary Information for
Elastic Mucus Strands Impair Mucociliary Clearance in Cystic Fibrosis Pigs

Maria I. Pino-Argumedo[1], Anthony J. Fischer[2], Brieanna M. Hilkin[1], Nicholas D. Gansemer[1], Patrick D. Allen[2], Eric A. Hoffman[3,4], David A. Stoltz[1,4,5], Michael J. Welsh[1,5,6], Mahmoud H. Abou Alaiwa[1,4]

Department of Internal Medicine[1],
Pappajohn Biomedical Institute,
Roy J and Lucille A Carver College of Medicine,
University of Iowa, Iowa City, Iowa 52242

Department of Pediatrics[2],
Pappajohn Biomedical Institute,
Roy J and Lucille A Carver College of Medicine,
University of Iowa, Iowa City, Iowa 52242

Department of Radiology[3],
Roy J and Lucille A Carver College of Medicine,
University of Iowa, Iowa City, Iowa 52242

Department of Biomedical Engineering[4],
University of Iowa, Iowa City, Iowa 52242

Department of Molecular Physiology and Biophysics[5],
Roy J and Lucille A Carver College of Medicine,
University of Iowa, Iowa City, Iowa 52242

Howard Hughes Medical Institute[6]
University of Iowa, Iowa City, Iowa 52242

Address Correspondence to: michael-welsh@uiowa.edu; mahmoud-aboualaiwa@uiowa.edu

This PDF file includes:

Supplementary Methods
Figures S1 to S2

Other supplementary materials for this manuscript include the following:

Videos 1 to 4

Supplementary Methods

Mucus microrheology. The volume of mucus that can be collected from the airways is small, which precludes the use of standard rheological techniques. We adapted a one-point microrheology technique to measure mucus biophysical properties *in vitro*. We collected mucus from a small tracheal window using sterile polyester tipped applicators (Puritan ®). From one animal, we can collect ~ 5- 25 μl of mucus. We embedded 1 μm fluorescent polystyrene microspheres in the sample and imaged in a small well (~ 5 μl mucus) created from a layer of paraffin sandwiched between a microscope slide and a coverslip. We recorded the motion of beads using a high-speed video camera attached to a Nikon A1R confocal with a 100x objective (NA1.4, oil) at 50 frames per second. We acquired 2 minute long videos from 10 different fields of view. We tracked the trajectory of each individual bead (~50-200 beads per field of view) in a 2 min long video using a tracking algorithm and microrheology tools for Interactive Data Language (IDL) written by John Crocker and Eric Weeks (http://www.physics.emory.edu/faculty/weeks//idl/rheo.html). We calculated the mean squared displacements of each individual bead and calculated the time-dependent complex shear modulus Gs and its viscoelastic moduli (Gp and Gpp) by the fluctuation-dissipation relationship as previously described (1-3).

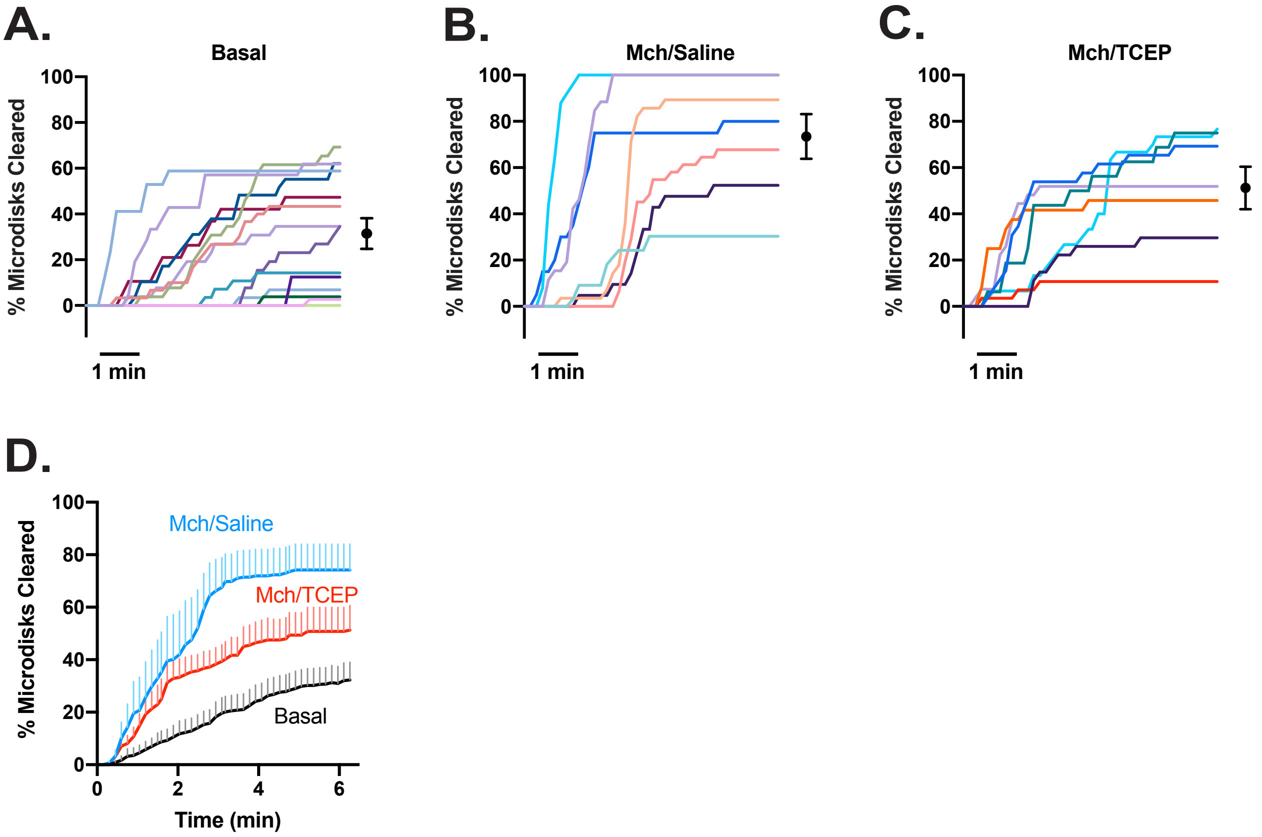

Suppl. Fig. 1. Microdisk clearance in non-CF pig airway. Data reproduced from Fischer et al(4). **(A, B & C).** % of microdisks cleared stratified by experimental condition. Each animal is depicted with a unique color. Conditions include: **(A)** basal unstimulated, **(B)** simultaneous IV methacholine and inhaled saline, and **(C)** simultaneous IV methacholine and inhaled TCEP. **D.** Mean percent of microdisks cleared from the field. Lines represent mean and standard error for basal (black), IV methacholine and inhaled saline (blue) or IV methacholine and TCEP (red).

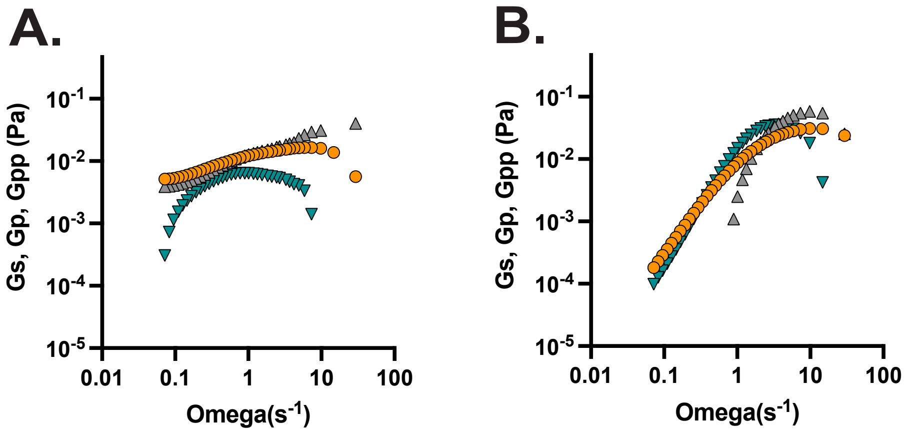

Suppl. Fig. 2. Microrheology of CF pig mucus. Data are time-dependent 1-point microrheology of mucus collected from CF pigs after **(A)** saline aerosolization, **(B)** TCEP aerosolization. Gs (complex modulus, orange, circle), Gp (storage or elastic modulus, gray, triangle), Gpp (loss or viscous modulus, teal, inverted triangle). Axes are log-scaled. Representative graph from 5 different biological replicates.

<u>**Supplementary Video # 1**</u>
Video 1.mp4
Movement of microdisks in CF pig airway under basal condition. Maximum intensity projection in coronal plane of a dynamic CT scan from a CF pig showing microdisk deposition and movement in the airways under basal unstimulated condition. Grayscale inverted to enhance contrast. Radiodense objects such as microdisks and bones will show in black. Microdisks travel towards the larynx (upward in the video) as they transport in the airway. Width = 85 mm. Time is compressed from 6.3 min

<u>**Supplementary Video # 2**</u>
Video 2.mp4
Movement of microdisks in CF pig airway under methacholine stimulated condition and aerosolized saline. Maximum intensity projection in coronal plane of a dynamic CT scan from a CF pig showing microdisk deposition and movement in the airways after stimulation with IV methacholine and aerosolized saline. Grayscale inverted to enhance contrast. Radiodense objects such as microdisks and bones will show in black. Microdisks travel towards the larynx (upward in the video) as they transport in the airway. Sometimes this forward movement is interrupted by a recoil and then the microdisks resume forward movement. Width = 60 mm. Time is compressed from 6.3 min

<u>**Supplementary Video # 3**</u>
Video 3.mp4
Retraction of a mucus strand. A confocal microscopy video with a 25x objective lens shows the trachea surface of a CF pig. Beating of cilia propels free particles upward. A mucus strand, labeled with red fluorescent nanospheres, abruptly retracts in the opposite direction of ciliary beating. Time is compressed from 80s; the last 5 sec in slow motion at 0.5x speed.

<u>**Supplementary Video # 4**</u>
Video 4.mp4
Cleavage of mucus strands by TCEP. Panoramic confocal microscopy with a 4x objective lens shows the trachea surface of a CF pig after methacholine stimulation. Mucus strands, identified by red fluorescent nanospheres and depicted here as white, are shown emerging from glands on the airway surface. Following addition of TCEP, the strands of mucus are cleaved. Distal portions of the strand are transported to the rostral edge of the tissue at top. Proximal fragments of these strands are retained at submucosal gland ducts. Image dimensions 8.5x14 mm. Time is compressed from 15.3 min

Supplementary References

1. J. C. Crocker, B. D. Hoffman, Multiple-particle tracking and two-point microrheology in cells. *Methods Cell Biol* **83**, 141-178 (2007).
2. D. B. Hill *et al.*, A biophysical basis for mucus solids concentration as a candidate biomarker for airways disease. *PLoS One* **9**, e87681 (2014).
3. D. B. Hill *et al.*, Pathological mucus and impaired mucus clearance in cystic fibrosis patients result from increased concentration, not altered pH. *Eur Respir J* **52** (2018).

4. A. J. Fischer *et al.*, Mucus strands from submucosal glands initiate mucociliary transport of large particles. *JCI Insight* **4** (2019).
